# Profiling microplastics in a forgotten river system in Southern Africa

**DOI:** 10.1007/s10661-025-13800-5

**Published:** 2025-03-04

**Authors:** Heinrich Theodor Jacob Dahms, Richard Greenfield

**Affiliations:** 1 Department of Zoology, University of Johannesburg, Kingsway Campus, Auckland Park, South Africa; 2 Institute for Alpine Environment, Eurac Research, Bolzano, Italy

**Keywords:** Velocity, Distribution, Water, Sediment, Polymers, Seasonality

## Abstract

Microplastics have been studied in rivers worldwide with far-reaching implications for aquatic ecosystems. What is less understood is how microplastics distribute through rivers, as microplastics do not distribute ubiquitously through a river system. This study described the microplastic profile of the Nyl, Mogalakwena and Limpopo Rivers in South Africa. The study aimed to determine the driving environmental factors of microplastic distribution in a river system over multiple seasons. The study discovered relationships between flow and microplastics in sediment. This relationship allowed microplastics to have significantly (*p* < 0.05) different distributions over seasons. Seasons with reduced flow had higher mean microplastic abundances in water (1436 ± 4492 particles.m^−3^) and sediment (1710 ± 4951 particles.kg^−1^dw), which decreased in both water (59 ± 46 particles.m^−3^) and sediment (17 ± 11 particles.kg^−1^dw) during the high flow season. Although microplastic abundances decreased with increased flow, a more homogenous distribution was detected through the entire system in the high flow period. The results suggest that microplastics could become trapped and increase significantly during reduced flow but become more evenly distributed during high flow seasons. The microplastics had also become bioavailable, being found in benthic macroinvertebrates in the river system at varying concentrations with a mean of 29 ± 33 particles.g^−1^ww, which could not be related to environmental matrices. Significant differences (*p* < 0.05) were determined between microplastic polymers found in water compared to sediment in all seasons. The study is the first in this transboundary river system, which impacts multiple African nations and a RAMSAR accredited wetland of international importance.

**Supplementary Information:**

The online version contains supplementary material available at 10.1007/s10661-025-13800-5.

## Introduction

The introduction of plastic into the environment might be one of the most significant human impacts on the environment over the last decade. The amount of plastic pollution that has globally been exerted on the environment has been so extensive that plastic pollution can replace fossils as an identifying factor for the Anthropocene (Alves et al., 2023; Rose et al., 2021). Plastic pollution has such an overreaching impact that it can be related to multiple UN Sustainable Development Goals, highlighting the need for more extensive research on this subject (Walker et al., 2021). Although larger plastics are easier to detect, unseen microplastics may present a greater environmental risk (Barboza et al., 2018; Guo & Wang, 2019; Li et al., 2018). Microplastics are small plastic particles (5–0.01 mm) classified as primary or secondary microplastics (Arthur et al., 2008; USEPA, 2024). Plastics such as tiny beads used in air blasting are examples of primary microplastics or plastics produced in smaller dimensions (Arthur et al., 2008; Li et al., 2018). Larger plastics broken down into smaller fractions are considered secondary microplastics (Arthur et al., 2008; Li et al., 2018). Microplastic research has grown significantly, and microplastics have been detected globally (Rochman et al., 2015; Li et al., 2018; Guo and Wang et al., 2019; Lu et al., 2022).

In African countries, microplastic research in freshwater aquatic environments lags behind developed countries (Alimi et al., 2021; Aragaw, 2021; Okeke et al., 2022). In the review by Aragaw et al. (2021), it was found that South Africa was the leading African nation regarding microplastic research in aquatic systems with 18 publications, followed by Nigeria with seven publications at the time the review was published (Weideman et al., 2019, 2020; Dahms et al., 2022; Ramaremisa et al., 2022; Saad et al., 2022A; 2022B). There is, therefore, a need for continued research of microplastics in Africa and South Africa, with microplastics in rivers being a critical area.

Although microplastic research has increased globally, how microplastics could distribute in rivers remains unanswered. It was initially believed that microplastics in rivers would migrate downstream into the oceans, with five rivers introducing 80% of ocean plastic pollution (Schmidt et al., 2017). The following study by Lebreton et al. (2017) proposed 47 rivers producing 80% of the ocean microplastics; however, more recently, Meijer et al. (2021) suggested that over 1656 rivers contribute to ocean plastic pollution. Rivers, however, are not a singular habitat and can vary greatly, as described in the River Continuum Concept (RCC) (Vannote, 1980; Dobbs and Maasri, 2022). This, in return, impacts instream and riparian vegetation, which will then impact the animal biodiversity found there (Dobbs and Maasri, 2022).

The following question would be how microplastics play a part in the RCC. Unlike a pollutant that would diffuse across a water body, microplastics remain solid and can break down into individual molecular polymer chains (Jansen, 2016). This means that the microplastics could be significantly impacted by the river characteristics itself (Dahms et al., 2020; Weidemann et al., 2020; Owowenu et al., 2023). Characteristics such as increased velocity could lead to increased microplastic counts in water; however, increased discharge can lead to reduced concentrations of microplastics (Lofty et al., 2025). Opposingly, other studies have found positive correlations between increased discharge and microplastic particles (Moses et al., 2023). These relationships are critical to assess and estimate microplastic concentrations and their impact on the environment. Surrounding land use activities have also been found to increase concentrations particularly between rural and urban areas (Wagner et al., 2019). All these components highlight that only when microplastics are investigated through the longitudinal profile of the river (River continuum concept (RCC)) can a more holistic reflection of the distribution and impact of microplastics on biota be determined.

This study aims to provide insight into how microplastics could distribute through a river continuum and how stream characteristics can impact microplastic abundances. This would provide insight into where rivers must be protected from plastic pollution and where biota might be more significantly impacted. It was hypothesised that (i) microplastics will be found within the water, sediment and benthic macroinvertebrates in the Nyl, Mogalakwena and Limpopo River System; (ii) Microplastic distribution can be correlated to river characteristics such as depth, velocity and sediment grain profiles. (iii) Microplastic shapes would not impact its distribution, and therefore no significant difference will be detected in microplastic shapes found in water compared to sediment. (iv) Microplastic polymers do not distribute ubiquitously and therefore a significant difference will be detected in microplastic polymers found in water compared to sediment. (v) Seasonal variation will significantly (*p* < 0.05) impact microplastic abundances through the river continuum.

## Method

### Site selection

**Fig. 1:**
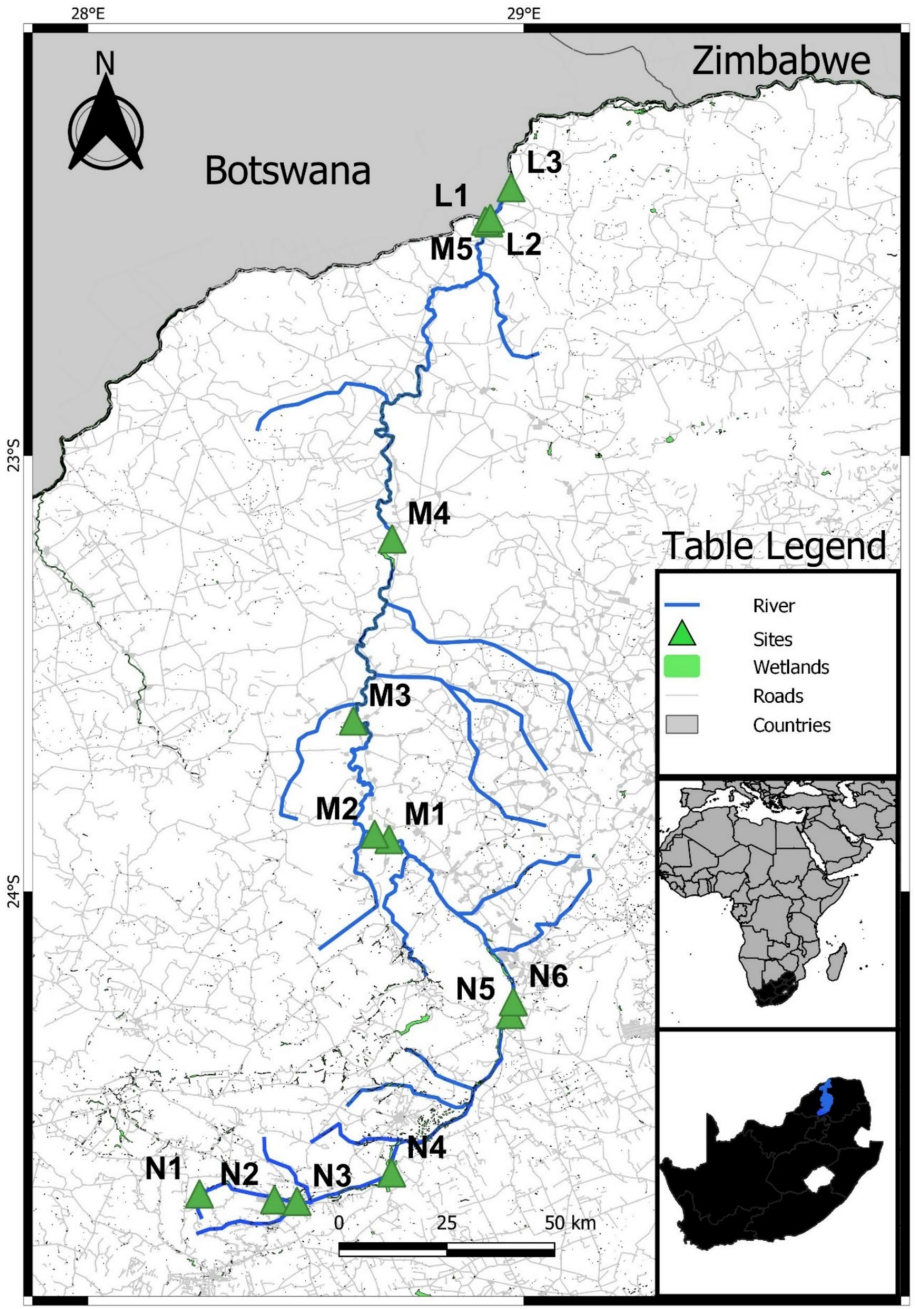
Map of the study area, the Nyl, Mogalakwena and Limpopo rivers and the investigated sites in Limpopo, South Africa. (N = Nyl River; M = Mogalakwena River; L = Limpopo River). Arrow indicates the general flow direction of the river

The study was conducted within three rivers that form one system in Limpopo, South Africa (Fig. 1). The system starts in the Nyl River, which begins in agricultural regions that passes through the large town of Modimolle (Dahms et al., 2017). Here, the Modimolle Sewage Treatment works significantly impact the river before it passes through a series of large wetlands, including the Nylsvley Wetland, a RAMSAR-accredited site (Baker & Greenfield, 2019; Dahms et al., 2017). The Nyl River then enters the town of Mokopane, where it flows into the confluence with the Mogalakwena River (Baker & Greenfield, 2019; Dahms et al., 2017). The Mogalakwena River, impacted by Mokopane, passes several large mines before passing some smaller rural villages and, finally, into nature reserves where little anthropogenic activities are found. The Mogalakwena River then flows into the confluence with the larger Limpopo River, a transboundary river, flowing past neighbouring countries Botswana, Zimbabwe, and Mozambique before entering the Indian Ocean (Nhassengo et al., 2021). The Mogalakwena River has been described as a non-perennial river, and the Limpopo River, which was a perennial river, has been described as a weak perennial river, with flow ceasing during parts of the year (Nhassengo et al., 2021). The river system has been described as critically impacted by anthropogenic activities; however, research on microplastic loads here has not been conducted (Greenfield et al., 2012; Dahms et al., 2017; Baker et al., 2019; Nhassengo et al., 2021). In this study, a total of 14 sites through the system were investigated (Fig. 1). Sampling occurred during three separate occasions named the low flow, high flow, and the intercept between these events, to determine how seasonal variation could impact the distribution of the microplastics (low flow=dry season; intercept=after first rainfalls; high=rainy season). In the low flow season, 11 sites were accessible, 12 sites were accessible during the intercept, and 11 sites in the high flow. This was due to some sites or matrices not being accessible during the various seasons. Time spent in the river at the sites were also limited due to the presence of crocodiles and hippos which posed a significant danger during sampling.

### Sample collection

#### *In situ* parameters

At eacated, the water velocity, depth, and *in situ* water quality parameters were measured. *In situ* water quality parameters (pH, Conductivity and Total Dissolved Solids-TDS) were measured using a handheld Eutech multi-probe water quality meter. Water velocity and depth were measured using a velocity head rod inserted into the river to measure the flow at the sampling point as described in the Rapid Habitat Assessment Methodology by DWAF (2009).

#### Water collection

A bulk water sample was filtered at each site to determine microplastic concentrations. A single replicate was collected at the most homogeneous point at each site due to time and safety concerns presented in the study area. At the point where velocity and depth were measured, large iron buckets were carefully inserted into the river, and care was taken to prevent the chance of dislodging any microplastics trapped in the sediment that would contaminate the sample. A bulk sample of 100 L of water was collected and filtered at the site through a series of Endecotts stainless steel wet washing sieves (Collicutt et al., 2019). The set consisted of 5000, 212 and 53-μm mesh size sieves to collect microplastics ranging from 5 to 0.05 mm in size. This prevented larger contaminants, such as plant debris, from being caught in the sieves. The sieves were then rinsed into glass jars three times with site water to collect the materials found in the sieves.

#### Sediment collection

At the sampling point after water was collected, sediment samples were taken. A single replicate of approximately 2 kg^−1^ of sediment was collected with a steel spade that was inserted approximately 15 cm^−1^ deep into the sediment (Coppock et al., 2017; Nel & Froneman, 2015). The sediment was scooped and placed in aluminium trays before being stored at room temperature. The sediment collected would be used for microplastic extraction and sediment grain size analysis.

#### Benthic macroinvertebrates

After all environmental samples were collected, invertebrates were sampled. Samples were collected using the kick-sweep-stir method with a 1-mm mesh size net (Klemm et al., 1990). The invertebrates in the net were then placed in an identification tray and identified at the family level using Aquatic Invertebrates of South African Rivers field guides (Gerber & Gabriel, 2002). In this study, Chironomid sp. larvae were collected at each site to represent microplastics ingested by biota (Nel et al., 2018). A total of 60 Chironomids were collected at each site; however, at sites where no Chironomids were found, Oligochaetes were collected to act as a representation of biota impacted by microplastics in the sediment (Nel et al., 2018). The invertebrates were immediately placed in 10% Neutrally Buffered Formalin (NBF), which would instantly kill the invertebrates before they could expel any gut content (Dahms et al., 2020; Klemm et al., 1990). Any other by-catch was safely placed back into the river.

### Laboratory analysis

#### Water microplastics extraction

The sampled materials collected in the field were placed into rinsed conical flasks for digestion. The volume of the water sample was measured, and an approximate amount of KOH was added to produce a 10% KOH solution (Joint Research Centre, 2014; GESAMP, 2019). The 10% KOH solution allowed for the digestion of organic matter but not any inorganic materials or microplastics that may have been present in the sample. The sample was left to stand for 24 h at room temperature befor

#### Sediment microplastics extraction

A sample of 500 g of wet-weight sediment was subsampled from the sediment and dried at 60°C for three days or until the weight no longer decreased, indicating that no more moisture was present in the sample. Microplastics were then extracted using five Sediment Microplastic Isolation units (SMIs) as described by Coppock et al. (2017). The SMIs constructed by Coppock et al. (2017) had microplastic recovery rates of approximately 95.5% and highlighted ZnCl_2_ as an inexpensive and accurate media to extract microplastics from sediment. In each SMI, a 50-g sample of sediment was added. The SMI was then filled with approximately 400 mL of ZnCl_2_ solution prepared at a 1:1 ratio of ZnCl_2_ and ultrapure Milli-Q water (Coppock et al., 2017). The solution would have a density of approximately 1.5 g.cm^−3^; this would allow for the collection of heavier plastic polymers, such as polyvinyl chloride, that other hypersaline solutions might be unable to dislodge (Coppock et al., 2017). A magnet was added to the SMI and placed on a magnetic stirrer. The sediment and ZnCl_2_ solution were stirred vigorously for 5 min to allow any microplastics trapped between sediment grains to be dislodged and rise through the solution to the top of the SMI. The SMI was then left to stand for 5 min, followed by another brief stir of 1 min and was then left to stand for 24 h to allow much finer sediment grains to settle at the bottom of the SMI (Coppock et al., 2017; Nel et al., 2018). The ball valve of the SMI was then closed, separating the bottom sediment and the less dense materials floating above the valve. The top solution was then vacuum filtered through a 20-μm filter paper. The filter papers were then placed in clean glass Petri dishes and dried in an oven, covered, for 24 h at 50°C to dry before visual identification by microscopy was conducted.

#### Sediment grain profiles

A 100-g subsample of dried sediment was used to determine the sediment grain profile of the points sampling was conducted. A series of Endecotts stainless steel sieves (4000, 2000, 500, 212, and 53 μm) was placed on a mechanical sediment shaker. The 100-g sediment sample was then inserted into the sieve system, which was shaken for 10 min. The sediment particles found in each sieve were weighed to determine the approximate percentage of particle sizes of the sediment (ASTM, 2000; USEPA, 2001). The sediment grain classification used by Cyrus et al. (2000) was then used to classify the sediment grain profiles at each site.

#### Microplastic extraction of macroinvertebrates

The macroinvertebrates were removed from the 10% NBF solution and rinsed with reverse osmosis water to remove the 10% NBF and external contamination from the net or tray used during sampling. The invertebrates were placed in groups of 10 individuals per sample and weighed (Dahms et al., 2020; Nel et al., 2018). After weighing, the organisms were washed and placed in Eppendorf tubes with a 10% KOH solution for 24 h (Nel et al., 2018). The organisms were then gently pressed with the blunt side of a dissection needle to gently open the exoskeleton and were vortexed to remove any microplastics trapped within the exoskeleton (Dahms et al., 2020).

### Microplastic identification

#### Visual identification by microscopy

Microplastics were identified using a stereoscopic dissection microscope with a magnification between 10× and 40×. Microplastics were then identified based on the guidelines created by Hidalgo-Ruz et al. (2012) and summarised in the Marine and Environmental Research Institute guidelines (MERI, 2015). A particle was only identified as a plastic when: no clear organic structure was seen; it did not break when pressed by a needle; it did not have a glass-like texture; fibres had to be evenly thick; every third plastic underwent the hot needle test where a hot needle is passed over the object and if it moved or curled it was identified as a plastic (Hidalgo-Ruz et al., 2012; MERI, 2015). Microplastics were then classified by colour and shape to determine where the plastics may have originated (Rochman et al., 2015; Windsor et al., 2019). Shapes used in this study were classified as filaments (fibres and fishing line), round (sphere and beads), angular (shards with clear lines and edges), and other shaped objects (foams, films, or particles with no clear dimensionsor margins) (MERI, 2015). Microplastic identification through visual methods may provide uneven results, and accuracy can be impacted by several aspects, such as the experience of the reader, the shape or colour of the material, and the size of the particle (Kotar et al., 2022). An interlaboratory study by De Frond et al. (2022), found microplastic visual identification as an accurate method for determining microplastics with a mean recovery range of 76% ± 10% (SE) for all sizes but a mean of 92% ± 12% (SD) was found for microplastics that were 20 μm and larger. In this study, microplastics smaller than 500 μm were heavily scrutinised before being counted as microplastics to reduce the risk of false identification.

#### Polymer identification by FT-IR analysis

To determine the polymers of the microplastics found in the river systems, a Shimadzu IRSpirit Fourier Transform Infrared Spectrophotometer (FT-IR) was used. Spectral analysis was conducted by the total reflectance mode with wavelengths of 4000 to 400 cm^−1^ spectral range with a resolution of eight. Each sample was scanned 32 times to provide a more accurate reading of the small microplastics and fibres found in this study. LabSolutions spectral software was used, and a complete material and polymer library was used to determine the 25 closest related materials to the particle. An accuracy score of 0.7 was required to positively identify a material, as described by Yang et al. (2015) and Migwi et al. (2020). The authors Yang et al. (2015) and Migwi et al. (2020) also allowed for the positive identification of polymers with a similarity score >0.6, which was also recorded in this study, but not used for the statistical analysis.

### Contamination control

To prevent contamination from materials used in this study, all equipment and glassware were cleaned by a soap and acid wash system (Giesy and Wiener, 1977). Before being used, all glassware and equipment were rinsed with reverse osmosis water to prevent contamination from airborne microplastic particles (Lusher et al., 2017). Plastic materials were used at a minimum, being replaced with glass or metal. During the digestion of samples, containers were kept sealed with aluminium foil to prevent contamination. Sediment Microplastic Isolation units may lead to contamination during use of the PVC due to the strenuous condition in which they are used (Nel et al., 2019). To prevent contamination, SMIs were cleaned and flushed before every use (Coppock et al., 2017; Nel et al., 2019B). Any material that may have resembled the SMI was excluded from sediment counts. During counting, microplastics were kept in clean glass Petri dishes that were permanently sealed, except when a plastic particle was removed and stored for further analysis. Movement in the laboratory was also restricted in the areas where microplastic analyses were conducted. In the field, the clothing of team members was noted, and negative controls were collected in the same containers used to store samples. Before samples were taken, all containers and equipment were cleaned before a sample was collected and sealed. To determine any possible contamination in the laboratory, negative controls of reverse osmosis water followed all the storage, digestion, density separation and counting steps. If any plastics were found, similar numbers and types of plastics would be subtracted from all samples. Only one other colour fibre was detected in the negative controls of the low flow water samples. No contamination was detected in the other negative controls in water, sediment, and invertebrates.

### Statistical analysis

Maps of the study area and distribution maps of microplastics in water and sediment of the seasons were drawn on QGIS 3.26.2. Bar graphs and tree maps were constructed in Microsoft Excel. Analysis of the results was conducted on SPSS v29. Descriptive statistics were conducted to determine the mean ± standard deviation (SD) of microplastic loads found in the system. The data was analysed to determine whether parametric or nonparametric testing was required through normality (Shapiro-Wilk test) and homogeneity of variance (Leven’s homogeneity of variance) testing. Mann-Whitney U tests were conducted to determine whether significant (p < 0.05) differences were found in the microplastic loads in the three matrices between seasons. Chi-squared tests were then used to determine whether any significant (*p* < 0.05) differences were found between the polymers found in water compared to sediment in each season and all seasons combined. Chi-squared tests were conducted to determine whether any significant (*p* < 0.05) differences were found between polymer types and shapes found in each season and all seasons combined. Multivariate analysis of environmental data and plastics was created on Canoco v5. A constrained ordination of environmental variables and microplastic shapes of each season was conducted through a redundancy analysis (RDA). An unconstrained ordination of polymer types and shapes was conducted through a Principal Component Analysis (PCA) to determine whether any relationship was found between polymer types and the shapes found in the study.

## Results

### Environmental parameters

#### *In situ* water quality parameters

Over all seasons sampled (Table S1), the pH ranged from 8.67 to 6.47, with a mean of 7.89 ± 0.58. During the individual seasons, the mean pH ranged from 8 ± 0.67 (low flow), 7.79 ± 0.74 (intercept), and 7.88 ± 0.27 (high flow). Conductivity ranged from 1509 μS.cm^−1^ to 44 μS.cm^−1^ with a mean over all seasons of 402 ± 346 μS.cm^−1^. Seasonal variation of conductivity was visible with means of 727 ± 403 μS.cm^−1^ (low flow), 255 ± 185 μS.cm^−1^ (intercept), and 240 ± 159 μS.cm^−1^ (high flow). The TDS ranged between 875 and 27 ppm over all seasons, with a mean of 238 ± 195 ppm. Similarly to pH and conductivity, seasonal variation impacted the mean TDS with means of 416 ± 231 ppm (low flow), 157 ± 104 ppm (intercept) and 146 ± 94 ppm (high flow). The water velocity of each site over each season was determined and recorded in Table S2. The maximum velocity measured was 0.65 m.s^−1^ with the lowest velocity <0.12 m.s^−1^. The maximum depth sampled in the study was 45 cm and the minimum depth 12 cm.

#### Sediment grain sizes

**Fig. 2:**
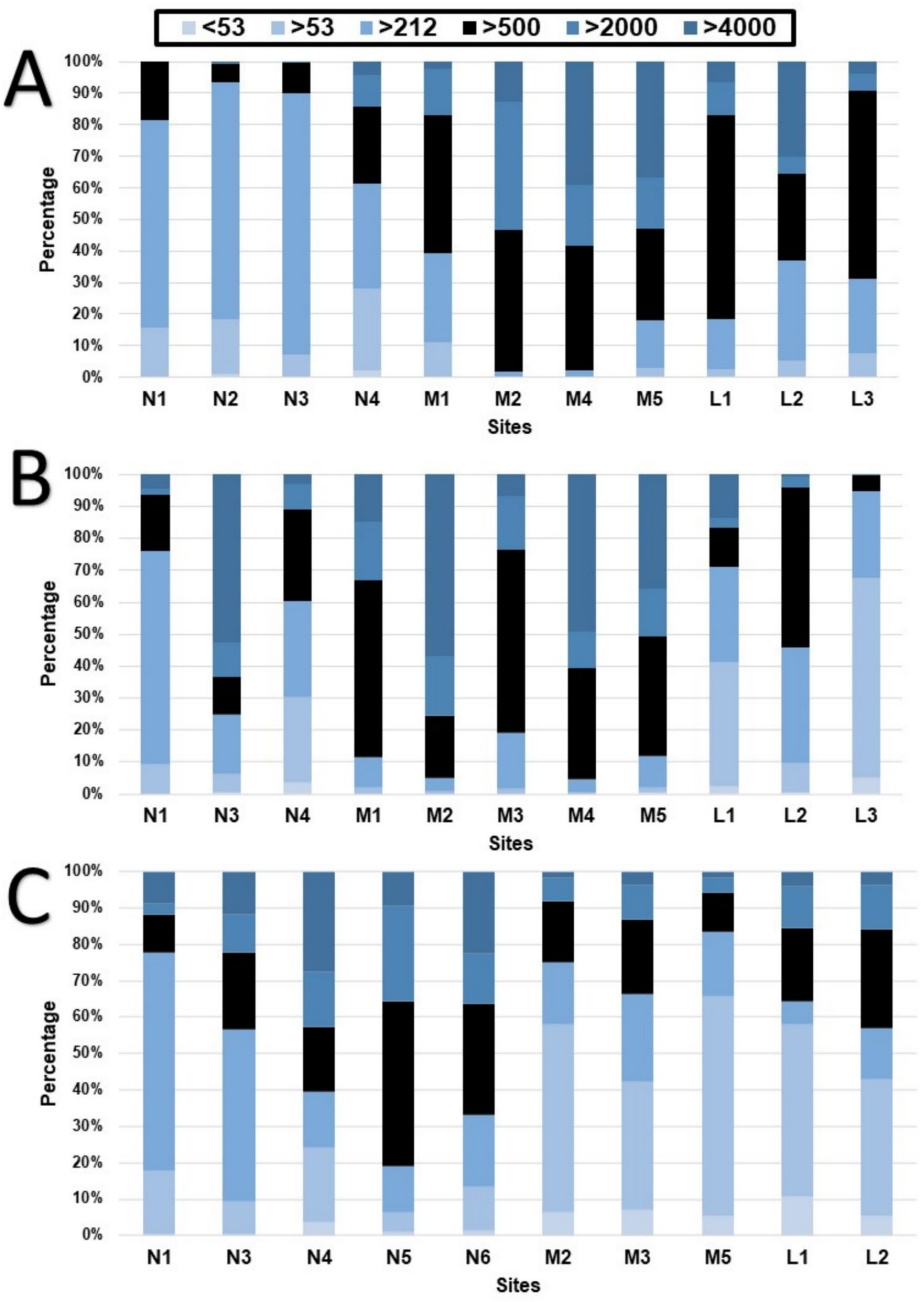
Stacked bar graphs of the percentage of the individual sediment particle sizes found in each site during the low flow (**A**), intercept (**B**), and high flow (**C**) seasons. Size fractions of particles are classified in the top legend

Sediment grain sizes varied between sites and seasons. The largest grain classification was gravel found at sites N3, M2, M4, and M5 (Table S2), with the finest composition being mud at M2, M3, M5, L1, L2, and L3. The high flow sediment profiles were expected to be much larger; however, they were dominated by very fine sand and mud found at 70% of the sites. The Nyl and Limpopo Rivers sediment grain profiles (Fig. 2) held finer sand and mud; however, the Mogalakwena River was characterised by more medium sand to gravel at the investigated site.

### Water microplastics

**Fig. 3:**
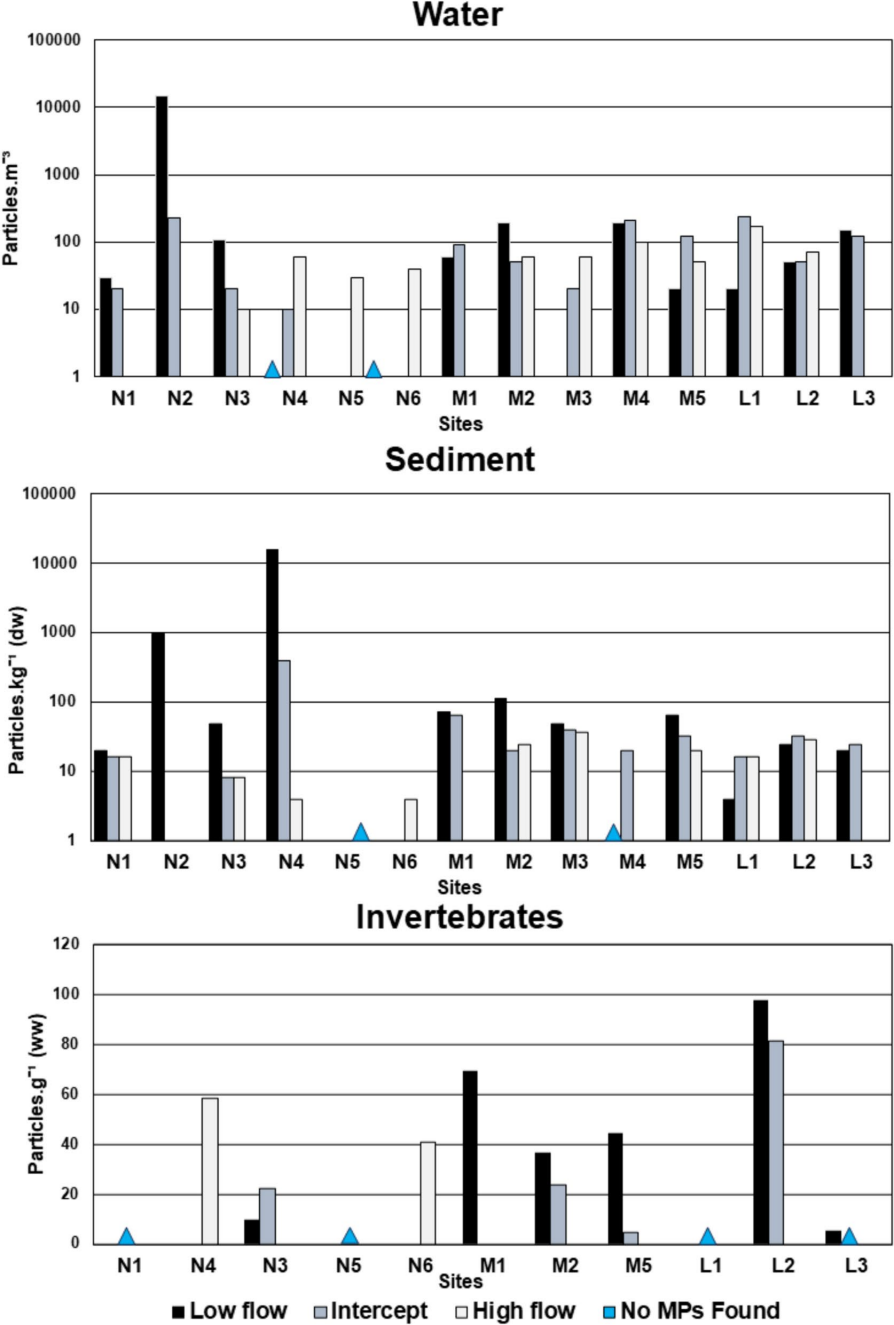
Bar graphs of microplastic loads found in water, sediment, and invertebrates over all three seasons

Water microplastics were collected in all samples excluding the N1 high flow and N4 low flow seasons, which had no microplastics (Fig. 3). The highest concentration at a single site found was N2 during the low flow season, with 14,980 particles.m^−3^ (Fig. 3). The overall mean of microplastics in water samples over all seasons was 518 ± 2556 particles.m^−3^. The low flow seasonal sampling had a mean of 1436 ± 4492 particles.m^−3^, the intercept a mean of 96 ± 90 particles.m^−3^, and the high flow a mean of 59 ± 46 particles.m^−3^. The most prevalent shape found across all seasons was other shaped microplastics (80.2%), followed by fibres as the second most prevalent (13.8%). However, if the significant amount of other shaped plastics found at N2 during the low flow season is removed, fibres are the most prevalent shape found (63.8%). Mann-Whitney *U* tests between microplastic loads in water over each season showed no significant (*p* > 0.05) difference between the seasonal events.

### Sediment microplastics

Microplastics were collected in all sediment samples across all seasons except in site M4 during the low flow season and M1 during the high flow season. Over all seasons, sediment microplastics had a mean of 545 ± 2,740 particles.kg^−1^dw (Fig. 3). The highest abundance of microplastics found in one site was in N4 during the low flow season with 15,777 particles.kg^−1^dw. The low flow season had the highest mean abundance of microplastics in sediment (1710 ± 4951 particles.kg^−1^dw), followed by the intercept (64 ± 119 particles.kg^−1^dw), and the high flow (17 ± 11 particles.kg^−1^dw) season having the lowest mean abundance of microplastics. The most prevalent microplastic shape found in sediment across all seasons were round (43.6%), followed by fibres (31%) and other shaped plastics (25.8%). An Independent sample t-test of the log-transformed data indicated significant (*p* < 0.05) differences between microplastic sediment loads between the low flow and high flow, and the intercept and high flow. No significant (*p* > 0.05) differences were found between the low flow and intercept microplastic loads.

### Invertebrate microplastics

The collection of Chironomid and Oligochaetes proved troublesome in the river system. Microplastics were only detected in 75% of the investigated groups (Fig. 3). The site with the highest abundance of ingested microplastics was L2, with a mean of 97.8 particles.g^−1^ww during the low flow season. The mean microplastic abundance over all seasons was 29 ± 33 particles.g^−1^ww. The mean microplastic abundances in the invertebrate groups were highest in the low flow season, 44 ± 35 particles.g^−1^ww, followed by the high flow mean of 33 ± 30 particles.g^−1^ww, and the lowest during the intercept, 18 ± 33 particles.g^−1^ww. Chironomid sp. larvae ingested 33 ± 36 particles.g^−1^ww and Oligochaetes 8.45 ± 8.42 particles.g^−1^ww. The most prevalent shape found in biota was fibres (95%), followed by other shape (3.27%) and angular (1.63%) microplastics the least prevalent shape.

### Polymer identification

**Fig. 4:**
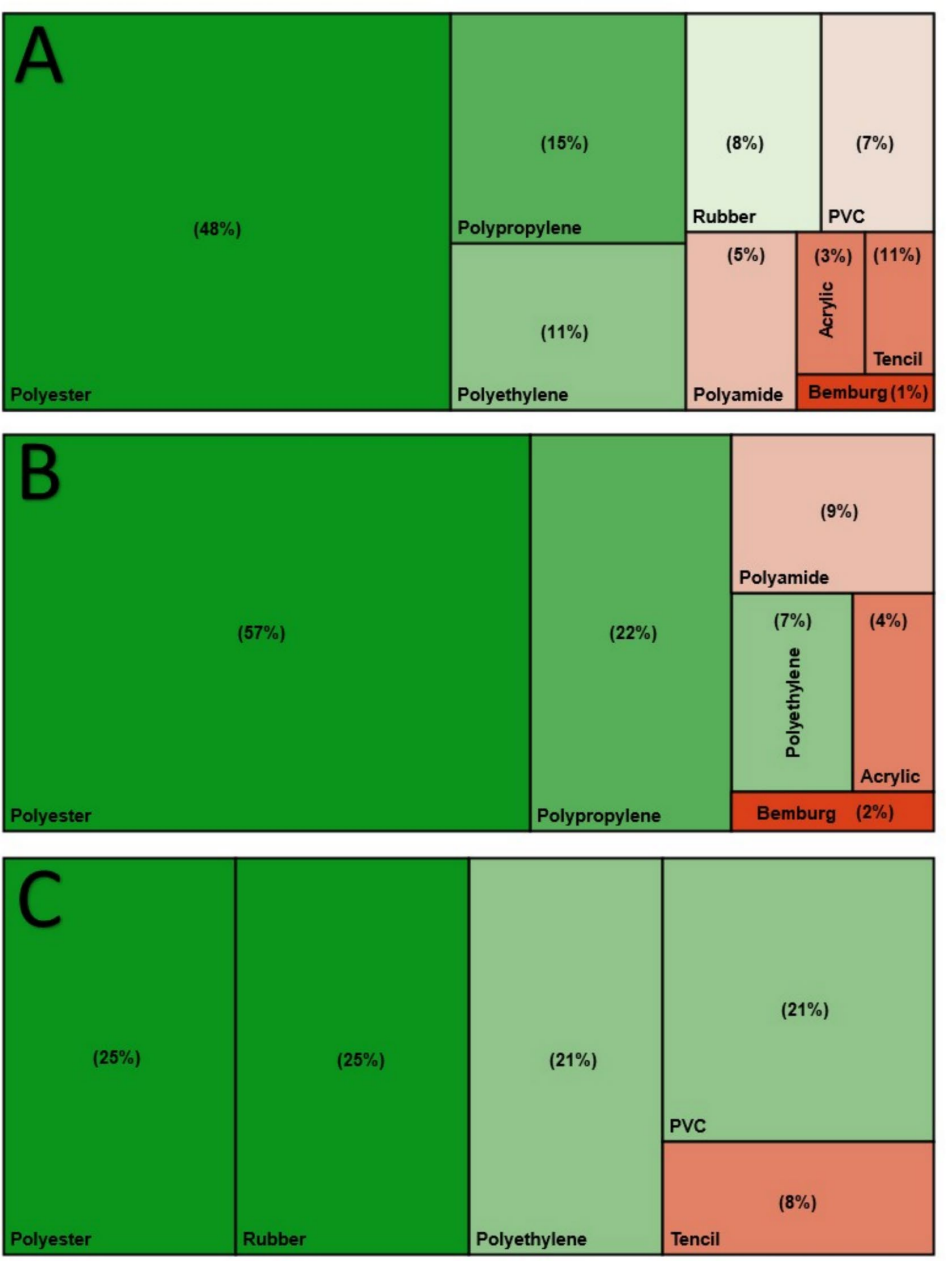
Tree maps of the percentages of polymer types found in all samples (**A**), water microplastics (**B**) and sediment microplastics (**C**)

Plastic particles were randomly selected and scanned on the FT-IR to determine their respective polymers. Of all the 225 scanned particles, 167 were closely related to plastic polymers, with 58 particles identified as none-plastic. This provides an accuracy of 74% for all particles regardless of the similarity score, which closely relates to the accuracy score (76 ± 10%) determined by De Frond et al. (2022). Of all the scanned particles, only 193 received a similarity score >0.6, and only 97 received an accuracy score >0.7. Of the particles with acceptable similarity scores (>0.7), 77% were identified as polymers. Nine primary polymer types were discovered in the study, with Polyester, Polypropylene, and Polyethylene being the most dominant polymers found (Supplementary materials: Fig. S1). Other noticeable polymers included Polyamide, Polyvinyl Chloride and Synthetic Rubber (Supplementary materials: Fig. S1). Different polymers (Fig. 4A) and polymer distributions were found in water (Fig. 4B) compared to sediment (Fig. 4C). Further analysis with chi-squared tests indicated significant differences (*p* < 0.05) between microplastic polymers found in water compared to sediment in each season and over all seasons combined.

### Multivariate analysis

**Fig. 5:**
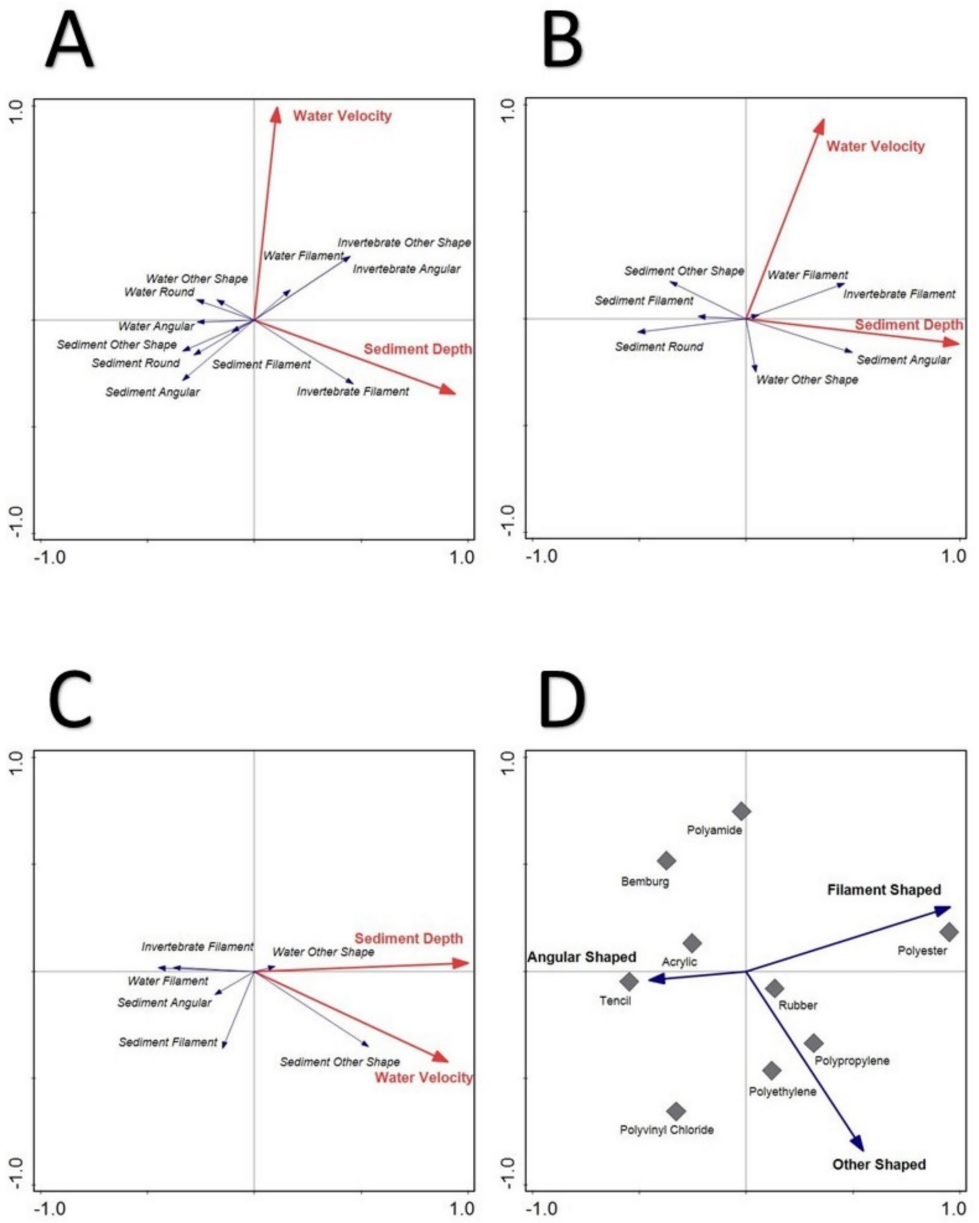
Multivariate analyses of microplastics and environmental variables through RDAs (graphs **A**- low flow, **B**- intercept and **C**- high flow), and a PCA (**D**) of microplastic shapes and polymers found in the study

Redundancy analysis of the relationship between environmental variables velocity and depth on microplastic types were conducted for each season (Fig. 5). In the RDA for the low flow event (Fig. 5A), 77.21% of the explained fitted cumulative variation falls on axis 1, with 22.79% of the variation on axis 2. The RDA for the intercept between seasons (Fig. 5B) had an explained fitted cumulative variation of 90.7% on axis 1 and 9.3% on axis 2. The RDA for the high flow season (Fig. 5C) had an explained fitted cumulative variation of 82.97% on axis 1, with the remaining 17.03% explained on axis 2. A Principal Component Analysis (PCA) of microplastic shapes and polymer types was conducted (Fig. 5D). The explained cumulative variation on axis 1 was 65.96% and 34.04 on axis 2 respectively.

## Discussion

Microplastics were detected for the first time within the Nyl, Mogalakwena and Limpopo Rivers. These rivers form a transboundary river system, with the microplastics detected in this study impacting South Africa and possibly its neighbouring countries, Botswana, Zimbabwe and Mozambique. This impact highlights the wide distribution that microplastics within a small section of a country could have on a global scale. The movement of microplastics needs to be better understood to understand how they might distribute within and impact ecosystems (Huang et al., 2020). The results of this study have aimed to determine how microplastics distribute from the point source, across the river continuum and over seasons.

Microplastics do not have a universal distribution through a system, with some areas having higher or lower microplastic concentrations (Owowenu et al., 2023). To determine whether these differences are related to the environment and not only anthropogenic activities, RDAs were produced (Fig. 5A–C). The RDAs indicated a relationship between velocity and sediment microplastics and mixed results between water microplastics and velocity. During the low flow season (Fig. 5A), the RDA indicated an inverse relationship with flow and sediment microplastics. This shows that when there are areas of reduced flow, microplastics may accumulate in the river’s sediment. Similarly, when there was an increase in flow, the water had higher microplastic counts. The relationship changed during the intercept (Fig. 5B). There was still an inverse relationship between velocity and sediment microplastics; however, the relationship between velocity and microplastics in the water decreased, showing an inverse relationship with other shaped microplastics in the water. In the high flow RDA (Fig. 5C), there was still an inverse relationship between microplastics in sediment and water velocity; however, this included water microplastics. The relationship between microplastics in sediment and velocity remained consistent through the seasons. This supports the theory that an influx of microplastics from the sediment into the water column could occur in areas or seasons where an increase in velocity occurs.

Sediment grain sizes were another environmental characteristic that impacted microplastic abundance in sediment. As previously detected by Dahms et al., (2020, 2022), areas with finer sediment profiles tend to have an increase in sediment microplastics. Areas with higher flow tend to have larger grain sizes, and reduced flow allows the sedimentation of sediment particles and microplastics in the water. The finer sediment then traps microplastics, which might be resuspended during events with increased flow. This relationship and the relationships in the RDAs were further investigated in the river system through seasonal changes.

**Fig. 6:**
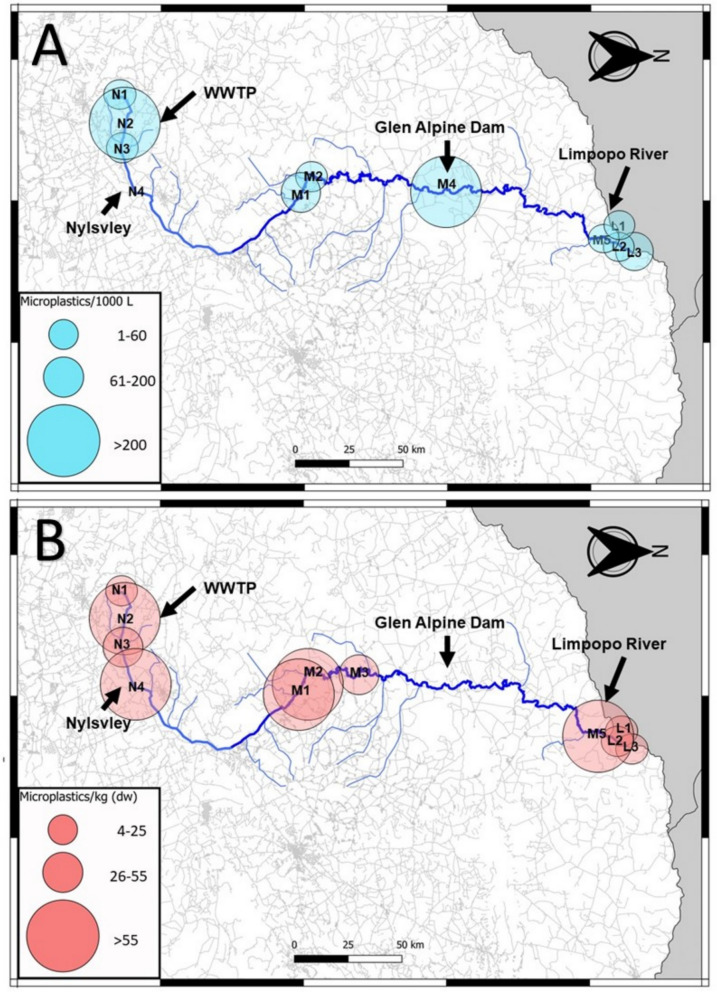
Distribution map of water microplastics (**A**) and sediment microplastics (**B**) found in the Nyl (N), Mogalakwena (M), and Limpopo (L) rivers during the low flow season

**Fig. 7:**
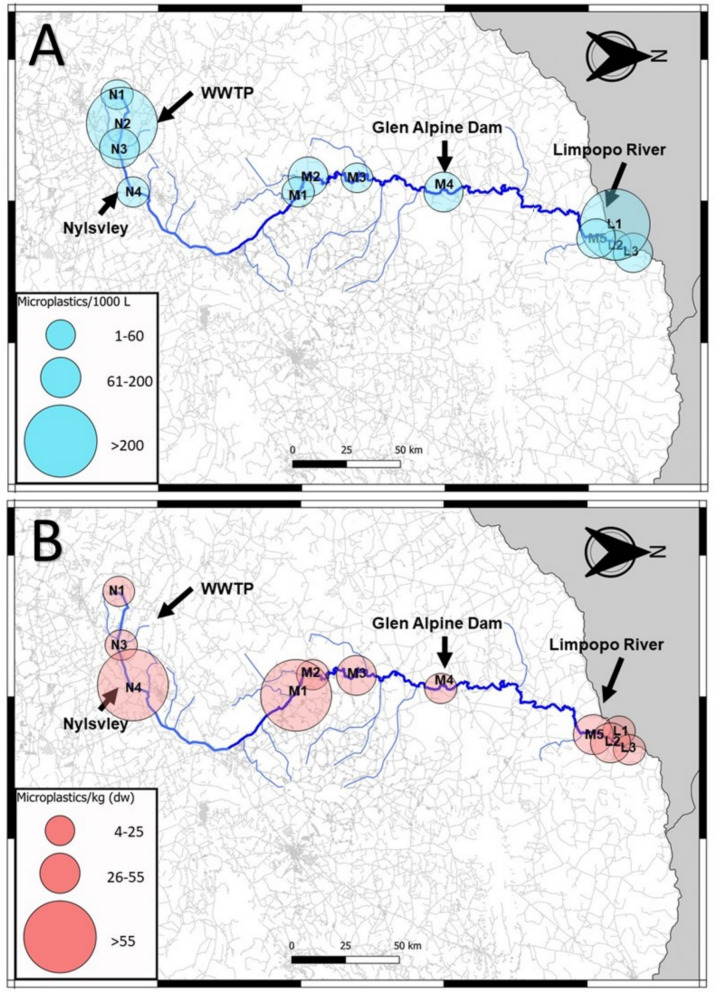
Distribution map of water microplastics (**A**) and sediment microplastics (**B**) found in the Nyl (N), Mogalakwena (M), and Limpopo (L) Rivers during the intercept season

**Fig. 8:**
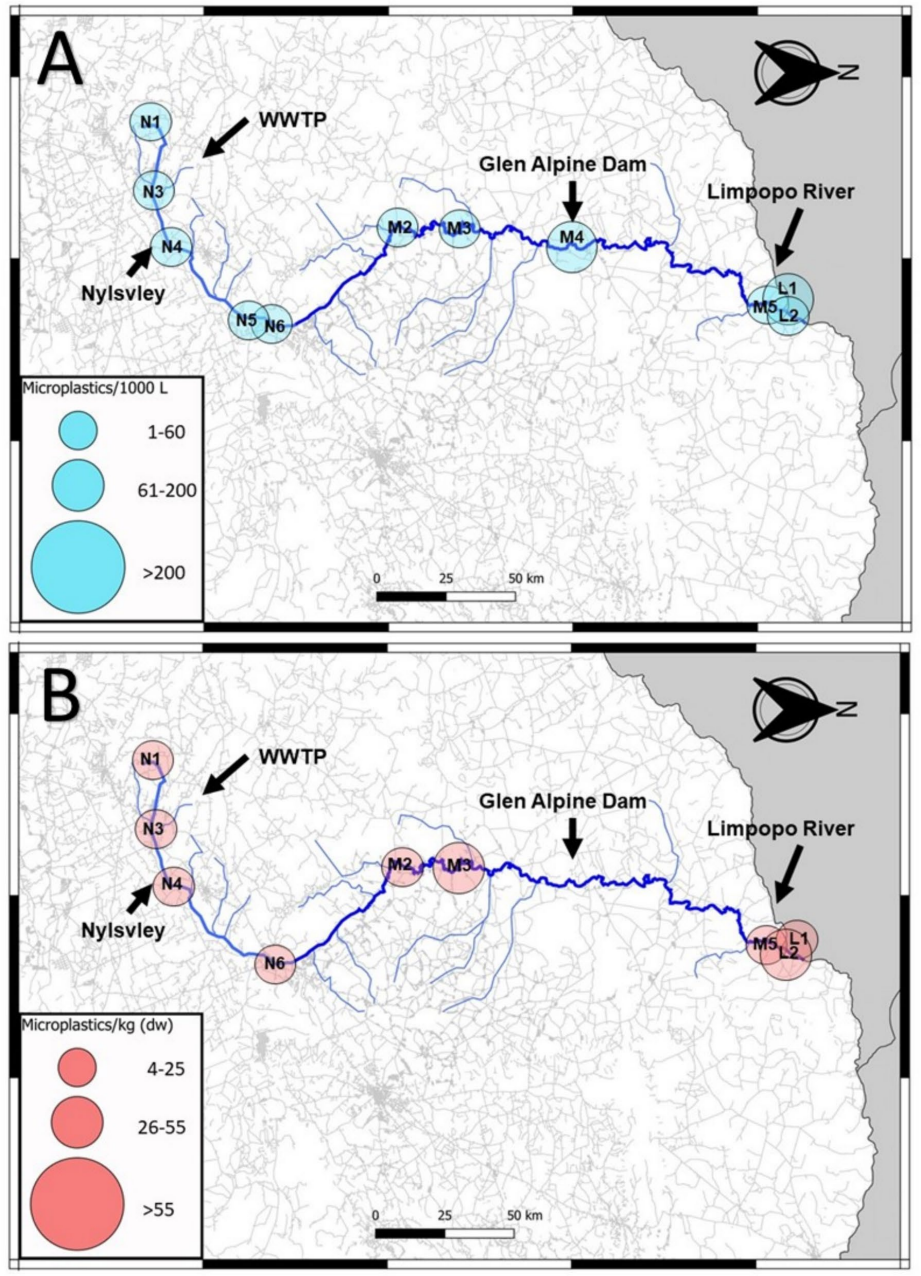
Distribution map of water microplastics (**A**) and sediment microplastics (**B**) found in the Nyl (N), Mogalakwena (M), and Limpopo (L) rivers during the high flow season

The movement of microplastics across the river continuum was investigated through distribution maps (Figs. 6, 7 and 8). Four primary sites of concern were identified in their roles as areas where an influx of microplastics could occur. Site N2 was an area of concern as it was located by a Waste-Water Treatment Plant (WWTP), where a significant increase of microplastics in water and sediment occurred (Figs. 6 and 7A). During the low flow season, the site was heavily polluted by sewage, which can be related to the poor water quality found here (Table S1). This site also had the highest individual microplastic concentration in water (14,980 particles.m^−3^) (Fig. 3). This influx of microplastics was still detected during the intercept (Fig. 7A); however, samples could not be collected during the high flow season. This reiterates the significant impact that WWTPs may have on microplastic loads in rivers globally (Sun et al., 2019). The river then continues through a series of large wetlands, including the next area of concern, site N4 the Nyl River Floodplain.

At N4, no microplastics were detected in the water during the low flow season (Fig. 6A); however, during that same season, the highest microplastic count in this study was detected in the sediment (15,777 particles.kg^−1^) (Fig. 3). Wetlands function as large filters that regulate flow in a river system; therefore, the reduced flow may have allowed microplastics to deposit and collect in the sediment, a relationship detected in all three RDAs conducted in this study (Fig. 5). It has been determined that even artificial wetlands can be used to reduce microplastic loads in water, which then increase within the sediment of the wetland (Lu et al., 2022). Whether the plants within the wetland or aquatic systems play a role in the deposition of microplastics has varied between studies, with more research on this required to determine its impact (Boshoff et al., 2023). As the seasons altered from the dry to the rainy season, more water was moving through the site, the microplastic concentration in the water started to increase during the intercept and high flow, similarly, the concentration of microplastics in the sediment decreased. This was also detected in the sediment grain profiles (Fig. 2), where larger sediment grains were detected in the intercept and high flow, compared to the low flow. The increase of water in the system may have allowed microplastics to resuspend into the water column, which could then move downstream.

The following site of concern was M4, located right after the Glen Alpine Dam wall. Dams have been known to act as sinks for microplastics, allowing it to increase in concentration and then deposit into the sediment of the dam (Dahms et al., 2022; Ramaremisa et al., 2022; Watkins et al., 2019). The site was a concern due to the massive influx of microplastics into the river after the dam wall (Fig. 6A). The site was characterised by large rapids and gravel, creating an environment perfect for resuspending and transferring microplastics collected in the dam, as no microplastics were found in the low flow sediment. This is again related to the previous relationships found in the RDAs (Fig. 5). As the seasons increased water in the system, the microplastic concentrations here decreased (Figs. 7 and 8).

The final areas of concern would be sites M5 and L1 to 3, which form the confluence between the Mogalakwena and Limpopo Rivers. The areas were investigated to determine whether the microplastics from the Mogalakwena River would increase the concentration of microplastics in the Limpopo River. During the low flow season (Fig. 6), the results indicated a minor increase of microplastics downstream of the confluence; however, the sediment microplastic loads in M5 were larger than the Limpopo River microplastic loads. These patterns changed during the intercept, with the Limpopo River contributing to higher microplastic concentrations in the water as flow increased in the system, which could have resuspended microplastics trapped in the fine sediment (Table S2). The sediment loads in the Mogalakwena reduced, indicating the microplastic may have become resuspended and transported downstream. The high flow continued the trend of lower microplastic loads in water and sediment within and after the confluence.

When the overall distribution through the river continuum is investigated, an apparent reduction in microplastics can be seen in the distribution maps of water and sediment over the three seasons as the water flow increased (Figs. 6, 7 and 8). A more ubiquitous distribution of microplastics is seen throughout the system during the high flow compared to the other seasons. The continuous reduction of microplastic loads in sediment highlights how the microplastics may have resuspended in times of increased flow (Dahms et al., 2020; Moses et al., 2023; Nel et al., 2018). This, however, is not reflected in the water samples where a general increase would be expected, but a decrease was also detected. The reduction could possibly be to the increased discharge in the high flow season, which has shown to either lead to an increased or decreased microplastic abundances (Lofty et al., 2025; Moses et al., 2023; Wagner et al., 2019). A new unit required for contextualizing microplastics according to discharge is required (Dahms and Greenfield 2024). This could indicate that microplastics may become more concentrated during reduced river flow in critical areas of concern, such as wetlands, WWTPs and dams. This would mean microplastics might have a greater impact on biota in these areas, being exposed to significantly higher concentrations of microplastics during these periods. However, it also indicates that these microplastics become resuspended when more water passes through the system during high flow seasons. Biota through the entire system might then be impacted and not in critical areas only.

When the differences in microplastic loads for the seasons were compared through an independent t-test, a significant difference (*p* > 0.05) could not be detected for water microplastics. However, a significant difference (*p* < 0.05) was discovered between microplastic loads in the sediment between the low and high flow and the intercept and high flow seasons. The significant differences (*p* < 0.05) between seasons and the relationships between flow and microplastic loads in sediment found in the RDAs (Fig. 5) indicate that these seasonal changes allow for more uniform microplastic distribution in high flow periods. The question remains whether the impact on biota would be greater in high flow periods with an even microplastic distribution or during low flow seasons with more localised areas of high microplastic concentrations. Unfortunately, no relationship could be found between microplastic loads in the invertebrates investigated in this study to the environment, and it is recommended that functional feeding groups or trophic levels should rather be investigated over a singular taxon.

The polymers that the microplastics might consist of, must be known to understand whether animals would be exposed to a singular group of polymers or to a variety of polymers. In this system, nine different polymers were discovered (Fig. 4A). Polymers comprise of different groups of monomers, and as the chain is altered, the characteristics of the polymer can change (Jansen, 2016). Polymers can therefore exist with a wide variety of densities which could then change the distribution of those plastics (Coppock et al., 2017; Owowenu et al., 2023). When the polymers of plastics found in this study were determined (similarity score >0.7), differences in the distributions between water and sediment were identified. A series of chi-squared analyses found a significant difference (*p* < 0.05) between polymers found in water compared to sediment. This relationship was further tested for each individual season with the same results. When all the particles with a similarity score of >0.6 were included, the chi-squared tests still indicated significant differences (*p* < 0.05) between polymers found in sediment compared to water. This could indicate that polymers do not have a universal distribution and may distribute according to their physical properties and not to the environment alone. The water column was dominated by polyester, polypropylene, and polyamide; however, the sediment was dominated by plastics such as synthetic rubber, polyethylene and polyvinyl chloride (Fig. 4B). This could indicate that animals found in the water column could be impacted differently by the plastics found there than those found in the sediment.

When the relationship between the polymers and the shapes was tested through a PCA (Fig. 5D), it was found that polyester and polyamide were more related to fibres and filaments. In contrast, polyethylene, polypropylene, polyvinyl chloride, and rubber were closely related to other shaped plastics. A chi-squared test then indicated significant differences (*p* < 0.05) between the shapes of plastics found in the water compared to the sediment, which relates to the polymers and where they distribute in the river. In this study, over 95% of microplastics in the invertebrates were fibres, indicating that polyester might significantly impact them. Polyester had the broadest distribution in this study, dominating the river system’s water column and sediment as fibres and filaments. This again highlights the importance of using the correct plastic polymer when conducting toxicological experiments on the impacts of microplastics on biota.

## Conclusion

To conclude, microplastics have been found for the first time in the Nyl, Mogalakwena, and Limpopo rivers, a transboundary river system of international importance. The microplastics in the river systems showed a complex distribution that can be impacted by various elements from anthropogenic activity, the environment or the characteristics of the microplastics themselves. The results highlighted how common river environments such as wetlands, dams, confluences, and WWTPs impact the distribution of microplastics. Physical characteristics of the river such as velocity, depth, particle size, plant life, and discharge could all lead to increasing or decreasing microplastic abundances. The results highlight how microplastics can distribute according to the RCC, leading to habitats with varying concentrations of microplastics within one river system. This could be applied globally to similar environments as areas of concern where humans and biota could be severely impacted. The hypothesis that (i) microplastics would be found in this river system was supported. The hypothesis that (ii) river characteristics such as depth, velocity and sediment grain sizes correlate to microplastic loads was supported in this study, as a relationship between velocity, microplastics and sediment grain profiles was seen throughout the river system. Hypothesis (iii) was rejected, as there was a significant (*p* < 0.05) difference between microplastic shapes found in water compared to sediment. The study also determined that polymers do not have a universal distribution as significant (*p* < 0.05) differences were found between polymer types in the water column compared to the sediment, allowing for the support of hypothesis iv. Although there were significant differences between microplastic loads and sediment over seasons (*p* < 0.05), hypothesis (v) could not be entirely supported as no statistically significant differences were found between the water microplastic concentrations over the seasons, therefore, the hypothesis is partially supported and requires further research.

## Supplementary Information

Below is the link to the electronic supplementary material.

ESM 1 (DOCX 189 KB)

## Data Availability

Data is provided within the manuscript or supplementary information files.

## References

[CR1] Alimi, O. S., Fadare, O. O., & Okoffo, E. D. (2021). Microplastics in African ecosystems: Current knowledge, abundance, associated contaminants, techniques, and research needs. *Sci. Tot. Env.,**755*, 1442422. 10.1016/j.scitotenv.2020.142422 10.1016/j.scitotenv.2020.142422 33011593

[CR2] Alves, F. L., Pinheiro, L. M., Bueno, C., Agostini, V. O., Perez, L., Fernandes, E. H. L., Weschenfelder, J., Leonhardt, A., Domingues, M., Pinho, G. L. L., & Garcia-Rodriguez, F. (2023). The use of microplastics as a reliable chronological marker of the Anthropocene onset in Southeastern South America. *Sci. Tot. Env.,**857*, 159633. 10.1016/j.scitotenv.2022.159633 10.1016/j.scitotenv.2022.159633 36280064

[CR3] ASTM, 2000. American Society for Testing and Materials Standards on Environmental Sampling. Standard practice for preparation of sediment samples for chemical analysis. Conshohocken, PA, USA. 163‒165.

[CR4] Aragaw, T. A. (2021). Microplastic pollution in African countries water systems: A review on findings, applied methods, characteristics, impacts and managements. *SN Appl. Sci.,**3*, 629. 10.1007/s42452-021-04619-z 34002166 10.1007/s42452-021-04619-z PMC8116826

[CR5] Arthur, C., Baker, J. and Bamford, H., 2008. International research workshop on the occurrence, effects, and fate of microplastic marine debris. In *Conference Proceedings of the International Research Workshop on the Occurrence, Effects, and Fate of Microplastic Marine Debris. Sept*. 9–11.

[CR6] Baker, N. J., & Greenfield, R. (2019). Shift happens: Changes to the diversity of riverine aquatic macroinvertebrate communities in response to sewage effluent runoff. *Ecological Indicators,**102*, 813–821. 10.1016/j.ecolind.2019.03.021

[CR7] Barboza, L. G. A., Vieira, L. R., Branco, V., Carvalho, C., & Guilhermino, L. (2018). Microplastics increase mercury bioconcentration in gills and bioaccumulation in the liver, and cause oxidative stress and damage in *Dicentrarchus labrax* juveniles. *Science and Reports,**8*, 1–9. 10.1038/s41598-018-34125-z 10.1038/s41598-018-34125-z PMC6199270 30353126

[CR8] Dahms, H.T:J., Greenfield, R., 2024. A review of the environments, biota, and methods used in microplastics research in South Africa. S. Afr. J. Sci. 120, 5/6, 10.17159/sajs.2024/16669

[CR9] Boshoff, B. J., Robinson, T. R., & von der Heyden, S. (2023). The role of seagrass meadows in the accumulation of microplastics: Insights from a South African estuary. *Marine Pollution Bulletin,**186*, 114403. 10.1016/j.marpolbul.2022.114403 36462418 10.1016/j.marpolbul.2022.114403

[CR10] Collicutt, B., Juanes, F., & Dudas, S. E. (2019). Microplastics in juvenile Chinook salmon and their nearshore environments on the east coast of Vancouver Island. *Environmental Pollution,**244*, 135–142. 10.1016/j.envpol.2018.09.137 30321708 10.1016/j.envpol.2018.09.137

[CR11] Coppock, R. L., Cole, M., Lindeque, P. K., Queirós, A. M., & Galloway, T. S. (2017). A small-scale, portable method for extracting microplastics from marine sediments. *Environmental Pollution,**230*, 829–837. 10.1016/j.envpol.2017.07.017 28734264 10.1016/j.envpol.2017.07.017

[CR12] Cyrus, D.P., Wepener, V., Mackay, C.F., Cilliers, P.M., Weerst, S.P., Viljoen, A., 2000. The effects of interbasin transfer on the hydrochemistry, benthic invertebrates and ichythyofauna of the Mhlathuze Estuary and Lake Nsezi. WRC Report No. 722/1/00, Water Research Commission, Pretoria, South Africa.

[CR13] Dahms, H. T. J., Tweddle, G. P., & Greenfield, R. (2022). Gastric microplastics in *Clarias gariepinus* of the upper Vaal River. *South Africa Front Environ Sci,**10*, 931073. 10.3389/fenvs.2022.931073

[CR14] Dahms, H. T. J., van Rensburg, G. J., & Greenfield, R. (2020). The microplastic profile of an urban African stream. *Science of the Total Environment,**731*, 138893. 10.1016/j.scitotenv.2020.138893 32408205 10.1016/j.scitotenv.2020.138893

[CR15] Dahms, S., Baker, N. J., & Greenfield, R. (2017). Ecological risk assessment of trace elements in sediment: A case study from Limpopo, South Africa. *Ecotoxicology and Environmental Safety,**135*, 106–114. 10.1016/j.ecoenv.2016.09.036 27721124 10.1016/j.ecoenv.2016.09.036

[CR16] De Frond, H., Hampton, L. T., Kotar, S., Gesulga, K., Matuch, C., Lao, W., Weisberg, S. B., Wong, C. S., & Rochman, C. M. (2022). Monitoring microplastics in drinking water: An interlaboratory study to inform effective methods for quantifying and characterizing microplastics. *Chemosphere,**298*, 134282. 10.1016/j.chemosphere.2022.134282 35283150 10.1016/j.chemosphere.2022.134282

[CR17] DWAF, 2009. Department of Water Affairs. Rapid habitat assessment model manual. Report no RDM/ Nat/00/CON/0707. Authors: D Louw & CJ Kleynhans Submitted by Water for Africa.

[CR18] Dodds, W. K., & Maasri, A. (2022). *The River Continuum Concept. Inland Waters.,**2*, 237–248. 10.1016/B978-0-12-819166-8.00105-5

[CR19] Gerber, A., Gabriel, M.J.M., 2002. Aquatic invertebrates of South African rivers, field guide. Institute for water quality studies. Department of Water Affairs and Forestry. First edition. Pretoria, South Africa.

[CR20] GESAMP. (2019). Joint group of experts on the scientific aspects of marine environmental protection. Guidelines for the monitoring and assessment of plastic litter and microplastics in the ocean. In Kershaw, P. J., Turra, A., & Galgani, F. (Eds.), (IMO/FAO/UNESCO-IOC/UNIDO/WMO/IAEA/UN/UNEP/UNDP/ISA). Rep Stud GESAMP No. 99, p 130.

[CR21] Giesy, J. P., & Wiener, J. G. (1977). Frequency distribution of trace metal concentrations in five freshwater fishes. *Transactions of the American Fisheries Society,**106*, 393–403. 10.1577/1548-8659(1977)106%3c393:FDOTMC%3e2.0.CO;2

[CR22] Greenfield, R., van Vuren, J. H. J., & Wepener, V. (2012). Heavy metal concentrations in the water of the Nyl River system, South Africa. *African Journal of Aquatic Science,**37*, 219–224. 10.2989/16085914.2011.653005

[CR23] Guo, X., & Wang, J. (2019). The chemical behaviors of microplastics in marine environment : A review. *Marine Pollution Bulletin,**142*, 1–14. 10.1016/j.marpolbul.2019.03.019 31232281 10.1016/j.marpolbul.2019.03.019

[CR24] Hidalgo-Ruz, V., Gutow, L., Thompson, R. C., & Thiel, M. (2012). Microplastics in the marine environment: A review of the methods used for identification and quantification. *Envronmental Sci. Technol.,**46*, 3060–3075. 10.1021/es2031505 10.1021/es2031505 22321064

[CR25] Huang, D., Tao, J., Cheng, M., Deng, R., Chen, S., Yin, L., Li, R., 2020. Microplastics and nanoplastics in the environment: Macroscopic transport and effect on creatures. J. Hazard. Mater. In Press, Journal Pre-proof, 124399. 10.1016/j.jhazmat.2020.124399 10.1016/j.jhazmat.2020.124399 33191019

[CR26] Jansen, J.A., 2016. [WWW Document]. Ascend. Plastics- It’s all about molecular structure. Plastic engineering. https://read.nxtbook.com/wiley/plasticsengineering/september2016/consultantscorner_plastics.html. (accessed 24–06–23).

[CR27] Klemm, D.J., Lewis, P.A., Fulk, F., Lazorchak, J.M., 1990. Macroinvertebrate field and laboratory methods for evaluating the biological integrity of surface waters. Aquatic biology branch and development and evaluation branch, quality assurance research division, environmental monitoring systems laboratory- Cincinnati. United States Environmental Protection Agency (EPA). EPA/600/4–90/030.

[CR28] Kotar, S., McNeish, R., Murphy-Hagan, C., Renick, V., Lee, C. T., Steele, C., Lusher, A., Moore, C., Minor, E., Schroeder, J., Helm, P., Rickabaugh, K., De Frond, H., Gesulga, K., Lao, W., Munno, K., Thornton Hampton, L. M., Weisberg, S. B., Wong, C. S., … Rochman, C. M. (2022). Quantitative assessment of visual microscopy as a tool for microplastic research: Recommendations for improving methods and reporting. *Chemosphere,**308*, 136449. 10.1016/j.chemosphere.2022.136449 36115477 10.1016/j.chemosphere.2022.136449 PMC11197986

[CR29] Lebreton, L. C. M., Van Der Zwet, J., Damsteeg, J. W., Slat, B., Andrady, A., & Reisser, B. (2017). Riverplastic emissions to the world’s oceans. *Nature Communications,**8*, 15611. 10.1038/ncomms15611 10.1038/ncomms15611 PMC5467230 28589961

[CR30] Li, J., Liu, H., & Paul Chen, J. (2018). Microplastics in freshwater systems: A review on occurrence, environmental effects, and methods for microplastics detection. *Water Research,**137*, 362–374. 10.1016/j.watres.2017.12.056 29580559 10.1016/j.watres.2017.12.056

[CR31] Lofty, J., Sorisio, G. S., Kelleher, L., Krause, S., Ouro, P., & Wilson, C. (2025). Hydrological and hydraulic drivers of microplastics in a rural river sourced from the UK’s largest opencast coal mine. *Environmental Pollution,**902*, 125722. 10.1016/j.envpol.2025.125722 10.1016/j.envpol.2025.125722 39828202

[CR32] Lu, H. C., Ziajahromi, S., Locke, A., Neale, P. A., & Leusch, F. D. L. (2022). Microplastics profile in constructed wetlands: Distribution, retention and implications. *Environmental Pollution,**313*, 120079. 10.1016/j.envpol.2022.120079 36064057 10.1016/j.envpol.2022.120079

[CR33] Lusher, A. L., Welden, N. A., Sobral, P., & Cole, M. (2017). Sampling, isolating and identifying microplastics ingested by fish and invertebrates. *Analytical Methods,**9*, 1346–1360. 10.1039/c6ay02415g

[CR34] Meijer, L. J. J., van Emmerik, T., van der Ent, R., Schmidt, C., & Lebreton, L. (2021). More than 1000 rivers account for 80% of global riverine plastic emissions into the ocean. *Science Advances,**7*, 1–13. 10.1126/sciadv.aaz5803 10.1126/sciadv.aaz5803 PMC8087412 33931460

[CR35] MERI. (2015). *Marine and Environmental Research Institute*. Guide to microplastic identification. University of Florida.

[CR36] Migwi, F. K., Ogunah, J. A., & Kiratu, J. M. (2020). Occurrence and spatial distribution of microplastics in surface waters of Lake Naivasha. *Kenya. Environ. Chem.,**39*, 765–774. 10.1002/etc.4677 10.1002/etc.4677 32004390

[CR37] Moses, S. R., Löder, M. G. J., Herrmann, F., & Laforsch, C. (2023). Seasonal variations of microplastic pollution in the German River Weser. *Science of the Total Environment,**902*, 166463. 10.1016/j.scitotenv.2023.166463 10.1016/j.scitotenv.2023.166463 37607635

[CR38] Nel, H. A., Dalu, T., & Wasserman, R. J. (2018). Sinks and sources: Assessing microplastic abundance in river sediment and deposit feeders in an Austral temperate urban river system. *Science of the Total Environment,**612*, 950–956. 10.1016/j.scitotenv.2017.08.298 28886547 10.1016/j.scitotenv.2017.08.298

[CR39] Nel, H. A., & Froneman, P. W. (2015). A quantitative analysis of microplastic pollution along the south-eastern coastline of South Africa. *Marine Pollution Bulletin,**101*, 274–279. 10.1016/j.marpolbul.2015.09.043 26433774 10.1016/j.marpolbul.2015.09.043

[CR40] Nel, H., Krause, S., Sambrook Smith, G. H., & Lynch, I. (2019). Simple yet effective modifications to the operation of the Sediment Isolation Microplastic unit to avoid polyvinyl chloride (PVC) contamination. *MethodsX,**6*, 2656–2661. 10.1016/j.mex.2019.11.007 31799134 10.1016/j.mex.2019.11.007 PMC6881676

[CR41] Nhassengo, O. S. Z., Somura, H., & Wolfe, J., III. (2021). Environmental flow sustainability in the lower Limpopo River Basin. *Mozambique J Hydrol Reg Stud,**36*, 100843. 10.1016/j.ejrh.2021.100843

[CR42] Okeke, E. S., Olagbaju, O. A., Okoye, C. O., Addey, C. I., Chukwudozie, K. I., Okoro, J. O., Deme, G. G., Ewusi-Mensah, D., Igun, E., Ejeromedoghene, O., Odii, E. C., Oderinde, O., Iloh, V. C., & Abesa, S. (2022). Microplastic burden in Africa: A review of occurrence, impacts, and sustainability potential of bioplastics. *Chem. Eng. J. Adv.,**12*, 100402. 10.1016/j.ceja.2022.100402

[CR43] Owowenu, E., Nnadozie, C., Akamagwuna, F. C., Noundou, X. S., Uku, J. E., & Odume, O. N. (2023). A critical review of environmental factors influencing the transport dynamics of microplastics in riverine systems: Implications for ecological studies. *Aquatic Ecology,**57*, 557–570. 10.1007/s10452-023-10029-7

[CR44] Joint Research Centre, 2014. Institute for environment and sustainability. Guidance on monitoring of marine litter in European seas. Publication office. https://data.europa.eu/doi/10.2788/99816

[CR45] Ramaremisa, G., Ndlovu, M., & Saad, D. (2022). Comparatice assessment of microplastics in surface waters and sediments of the Vaal River, South Africa: Abundance, composition, and sources. *Environmental Toxicology and Chemistry,**41*, 3029–3040. 10.1002/etc.5482 36341489 10.1002/etc.5482 PMC9828735

[CR46] Rochman, C. M., Tahir, A., Williams, S. L., Baxa, D. V., Lam, R., Miller, J. T., Teh, F., Werorilangi, S., & Teh, S. J. (2015). Anthropogenic debris in seafood : Plastic debris and fibers from textiles in fish and bivalves sold for human consumption. *Science and Reports,**5*, 14340. 10.1038/srep14340 10.1038/srep14340 PMC4585829 26399762

[CR47] Rose, N.L., Turner, S.D., Unger, L.E., Curtis, C.J., 2021. The chronostratigraphy of the Anthropocene in southern Africa: Current status and potential. S. Afr. J. Geol. 124, 1093- 1106. 10.25131/sajg.124.0053

[CR48] Saad (A), D., Chauke, P., Cukrowska, E., Richards, H., Nikiema, J., Chimuka, L., Tutu, H.,. (2022). First biomonitoring of microplastic pollution in the Vaal River using Carp fish (*Cyprinus carpio*) “as a bio-indicator.” *Science of the Total Environment,**836*, 155623. 10.1016/j.scitotenv.2022.155623 10.1016/j.scitotenv.2022.155623 35508237

[CR49] Saad (B), D., Ndlovu, M., Ramaremisa, G., Tutu, H.,. (2022). Microplastics in freshwater: The first evaluation in sediment of the Vaal River. *South Africa. Heliyon.,**8*, e11118. 10.1016/j.heliyon.2022.e11118 10.1016/j.heliyon.2022.e11118 PMC9634034 36339993

[CR50] Schmidt, C., Krauth, T., Wagner, S., 2017 Export of plastic debris by rivers into the sea. Environ. Sci. Technol. 51, 12246– 12253. https://doi/10.1021/acs.est.7b02368 10.1021/acs.est.7b02368 29019247

[CR51] Sun, J., Dai, X., Wang, Q., van Loosdrecht, M. C. M., & Ni, B. J. (2019). Microplastics in wastewater treatment plants: Detection, occurrence and removal. *Water Research,**152*, 21–37. 10.1016/j.watres.2018.12.050 30660095 10.1016/j.watres.2018.12.050

[CR52] USEPA, 2024. United States Environmental Protection Agency. Microplastics Research. Water Research. https://www.epa.gov/water-research/microplastics-research#:~:text=EPA%20researchers%20define%20microplastics%2C%20or,is%20about%2080%2C000%20nanometers%20wide. (Accessed: 27/01/25).

[CR53] USEPA, 2001. United States Environmental Protection Agency. Methods for collection, storage and manipulation of sediments for chemical and toxicological analyses: Technical manual. EPA 823-B-01–002. United States Environmental Protection Agency, Office of Water, Washington DC, USA. Appendix G: 194‒208.

[CR54] Vannote, R. L., & MINSHALL, G.W., Cummins, K.W., Sedell, J.R., Cushing, C.E.,. (1980). The river continuum concept. *Canadian Journal of Fisheries and Aquatic Sciences,**37*, 130–137. 10.1139/f80-017

[CR55] Wagner, S., Klöckner, P., Stier, B., Römer, M., Seiwert, B., Reemtsma, T., & Schmidt, C. (2019). Relationship between discharge and river plastic concentrations in a rural and an urban catchment. *Environ Sci. & Tech.,**53*(17), 10082–10091. 10.1021/acs.est.9b03048 10.1021/acs.est.9b03048 31380631

[CR56] Walker, T. R. (2021). (Micro)plastics and the UN Sustainable Development Goals. *Curr Opin Green Sustain Chem,**30*, 100497. 10.1016/j.cogsc.2021.100497

[CR57] Watkins, L., Mcgrattan, S., Sullivan, P. J., & Walter, M. T. (2019). The effect of dams on river transport of microplastic pollution. *Science of the Total Environment,**664*, 834–840. 10.1016/j.scitotenv.2019.02.028 30769307 10.1016/j.scitotenv.2019.02.028

[CR58] Weideman, E. A., Perold, V., & Ryan, P. G. (2019). Little evidence that dams in the Orange– Vaal River system trap floating microplastics or microfibres. *Marine Pollution Bulletin,**149*, 110664. 10.1016/j.marpolbul.2019.110664

[CR59] Weideman, E. A., Perold, V., & Ryan, P. G. (2020). Limited long-distance transport of plastic pollution by the Orange-Vaal River system. *South Africa. Sci. Total Environ.,**727*, 138653. 10.1016/j.scitotenv.2020.138653 32325317 10.1016/j.scitotenv.2020.138653

[CR60] Windsor, F. M., Tilley, R. M., Tyler, C. R., & Ormerod, S. J. (2019). Microplastic ingestion by riverine macroinvertebrates. *Science of the Total Environment,**646*, 68–74. 10.1016/j.scitotenv.2018.07.271 30048870 10.1016/j.scitotenv.2018.07.271

[CR61] Yang, D., Shi, H., Li, L., Li, J., Jabeen, K., & Kolandhasamy, P. (2015). Microplastic pollution in table salts from China. *Environmental Science and Technology,**49*, 13622–13627. 10.1021/acs.est.5b03163 26486565 10.1021/acs.est.5b03163

