# Supplementary material for: Profiling microplastics in a forgotten river system in Southern Africa
Source: Environ Monit Assess. 2025 Mar 4;197(4):351. doi: 10.1007/s10661-025-13800-5 (PMC11880085; doi:10.1007/s10661-025-13800-5)
Supplement: Supplementary file 1 — (DOCX 189 KB) [file 10661_2025_13800_MOESM1_ESM.docx]

**
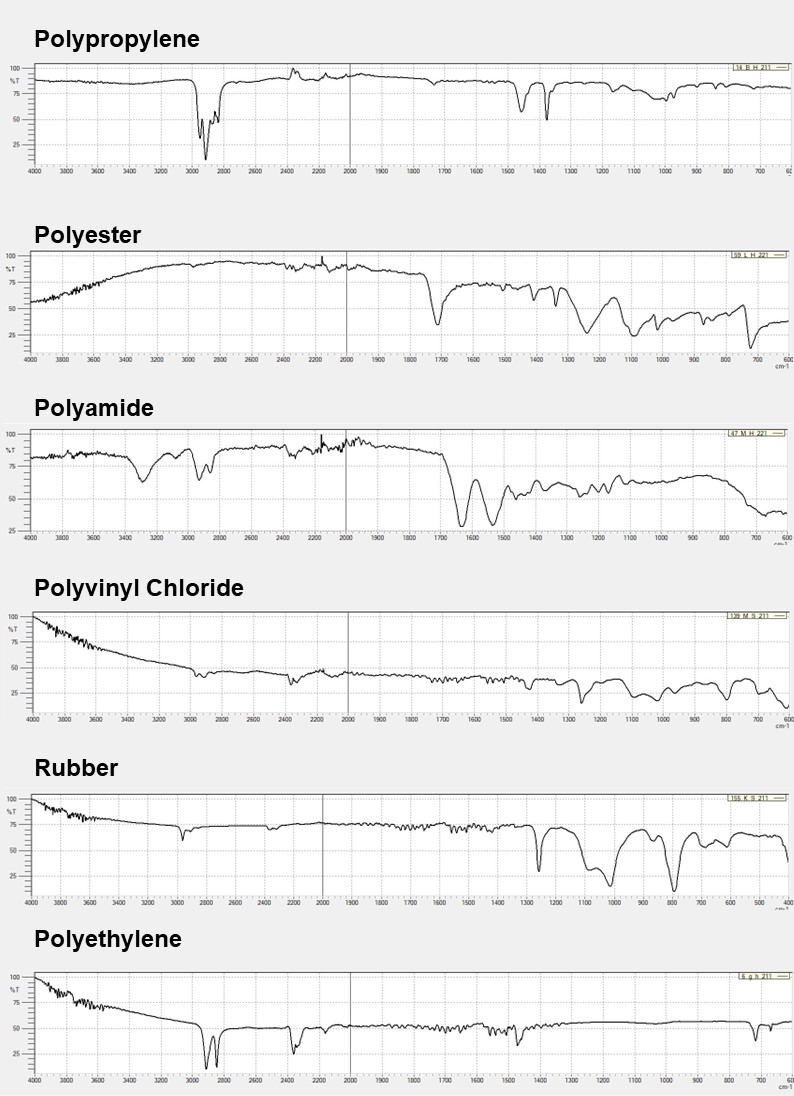
Supplementary materials**

**Fig S1**: FT-IR wavelengths of the six most prominent polymers found

**Table S1: *In-situ* water parameters pH. Conductivity. and Total Dissolved Solids of the investigated sites**

| **Site** |  | **pH** |  |  | **Conductivity** |  |  | **Total Dissolved Solids**  **(ppm)** |  |
| --- | --- | --- | --- | --- | --- | --- | --- | --- | --- |
|  |  |  |  |  | **(μS.cm^-1^)** |  |  |  |  |
|  | **Low flow** | **Intercept** | **High flow** | **Low flow** | **Intercept** | **High flow** | **Low flow** | **Intercept** | **High flow** |
| **N1** | 7.29 | 7.2 | 7.83 | 558 | 44.1 | 56.8 | 332 | 27.4 | 34.9 |
| **N2** | 7.21 | 7.37 |  | 1017 | 145.2 |  | 590 | 95.5 |  |
| **N3** | 7.34 | 6.47 | 7.63 | 697 | 158.1 | 137.5 | 404 | 98.4 | 86 |
| **N4** | 7.41 | 6.97 | 7.32 | 288 | 178.9 | 133.7 | 151 | 111 | 88.5 |
| **N5** |  |  | 7.79 |  |  | 248 |  |  | 154 |
| **N6** |  |  | 7.75 |  |  | 166.6 |  |  | 104 |
| **M1** | 8.58 | 7.27 |  | 235 | 132.1 |  | 137 | 81.9 |  |
| **M2** | 8.07 | 8.1 | 8.2 | 442 | 143.3 | 467 | 253 | 88.7 | 255 |
| **M3** |  | 8.53 | 8.27 |  | 169.6 | 489 |  | 104 | 307 |
| **M4** | 7.65 | 7.52 | 7.84 | 326 | 108.5 | 131.4 | 190 | 118 | 81.5 |
| **M5** | 8.37 | 8.32 | 7.99 | 1509 | 392 | 163.9 | 875 | 244 | 103 |
| **L1** | 8.77 | 8.44 | 8.07 | 1078 | 530 | 476 | 571 | 265 | 294 |
| **L2** | 9.17 | 8.67 | 8.05 | 822 | 531 | 167.5 | 477 | 327 | 102 |
| **L3** | 8.22 | 8.66 |  | 1026 | 531 |  | 596 | 329 |  |
|  |  |  |  |  |  |  |  |  |  |

**Table S2: Water velocity. Sediment depth. and Sediment grain classification of the investigated sites**

| **Site** |  | **Water velocity (m.sˉ¹)** |  |  | **Sediment depth (cmˉ¹)** |  |  | **Sediment grain classification**  **Cyrus et al. (2000)** |  |
| --- | --- | --- | --- | --- | --- | --- | --- | --- | --- |
|  | **Low flow** | **Intercept** | **High flow** | **Low flow** | **Intercept** | **High flow** | **Low flow** | **Intercept** | **High flow** |
| **N1** | 0.41 | 0.41 | 0.41 | 18 | 20 | 38 | Very fine sand | Very fine sand | Very fine sand |
| **N2** | 0.12 |  |  | 37.75 |  |  | Very fine sand |  |  |
| **N3** | 0.24 | 0.65 | 0.65 | 22.5 | 29 | 42 | Very fine sand | Gravel | Very fine sand |
| **N4** | <0.12 | <0.12 | <0.12 | 12 | 25 | 29 | Very fine sand | Very fine sand | Coarse sand |
| **N5** |  |  | <0.12 |  |  | 26 |  |  | Coarse sand |
| **N6** |  |  | <0.12 |  |  | 19 |  |  | Medium sand |
| **M1** | <0.12 | 0.12 |  | 37 | 18 |  | Medium sand | Coarse sand |  |
| **M2** | <0.12 | 0.41 | 0.48 | 30 | 21 | 45 | Very coarse sand | Gravel | Mud |
| **M3** |  | 0.12 | 0.24 |  | 25 | 26 |  | Coarse sand | Mud |
| **M4** | 0.24 | 0.12 |  | 45 | 26 |  | Very coarse sand/Gravel | Very coarse sand |  |
| **M5** | <0.12 | 0.24 | 0.12 | 21 | 28 | 24 | Very coarse sand/Gravel | Very coarse sand | Mud |
| **L1** | <0.12 | 0.12 | 0.12 | 34 | 21 | 24 | Coarse/Medium sand | Very fine sand | Mud |
| **L2** | <0.12 | 0.12 | <0.12 | 33 | 23 | 18 | Medium sand | Medium sand | Mud |
| **L3** | <0.12 | 0.24 |  | 21 | 32 |  | Medium/Fine sand | Mud |  |
